# miR-927 Regulates Photoreceptor Subtype Specification Through Yorkie and Sensory Opsins in *Drosophila*

**DOI:** 10.3390/cells15090841

**Published:** 2026-05-04

**Authors:** Hongli Ji, Shisong Zhang, Huiru Lu, Ruying Ma, Fengjie Xin, Jialin Che, Hui Wu, Gang Wang, Baotong Xie

**Affiliations:** 1 School of Life Sciences and Technology, Shandong Second Medical University, Weifang 261053, China; hlijih@163.com (H.J.); shisongz@126.com (S.Z.); hruluk@126.com (H.L.); ryingma@126.com (R.M.); 20240046@stu.sdsmu.edu.cn (F.X.); 20237140010@stu.sdsmu.edu.cn (J.C.); 2 Department of Integrative Biomedical & Diagnostic Sciences, School of Dentistry, Oregon Health and Science University, Portland, OR 97239, USA; wuhu@ohsu.edu

**Keywords:** *Drosophila*, miR-927, Yorkie, Rhodopsin 5, Hippo pathway

## Abstract

**Highlights:**

**What are the main findings?**

- miR-927 promotes yR8 photoreceptor identity while repressing pR8 fate in *Drosophila*.
- miR-927 may directly target both the Hippo pathway effector *Yorkie* and the terminal differentiation gene *Rh5* through their 3′UTRs.

**What are the implications of the main findings?**

- Coordinated repression of a signaling effector and its downstream target links Hippo pathway output to terminal gene expression.
- MicroRNAs can be integrated into bistable signaling networks to bias and stabilize binary cell fate decisions.

**Abstract:**

Binary cell fate decisions in the *Drosophila* retina generate R8 photoreceptor subtypes that express either blue-sensitive Rh5 or green-sensitive Rh6 opsins. These choices are governed by a Hippo pathway–dependent bistable switch, yet the mechanisms that couple pathway output to terminal opsin expression remain unclear. Here, we identify miR-927 as a regulator that biases R8 subtype fate. Loss of miR-927 increases Rh5-positive pR8 cells, whereas its overexpression promotes Rh6-positive yR8 identity. Mechanistically, miR-927 directly represses the terminal differentiation gene *Rh5* and is capable of repressing the Hippo pathway effector *yki* through its 3′UTR. This dual targeting couples pathway output to terminal gene expression, providing a mechanism to bias and stabilize subtype identity. More broadly, our findings illustrate how microRNAs can be integrated into bistable signaling networks to modulate binary cell fate decisions.

## 1. Introduction

Color vision relies on the ability of photoreceptors to detect distinct wavelengths of light [1,2,3]. In the compound eye of *Drosophila melanogaster*, this function is achieved through the precise specification of photoreceptor subtypes within each ommatidium, the repeating unit of the retina [4,5]. Each ommatidium contains eight photoreceptors, including the inner R7 and R8 cells that function together to mediate color vision [6,7,8,9].

**Figure 1:**
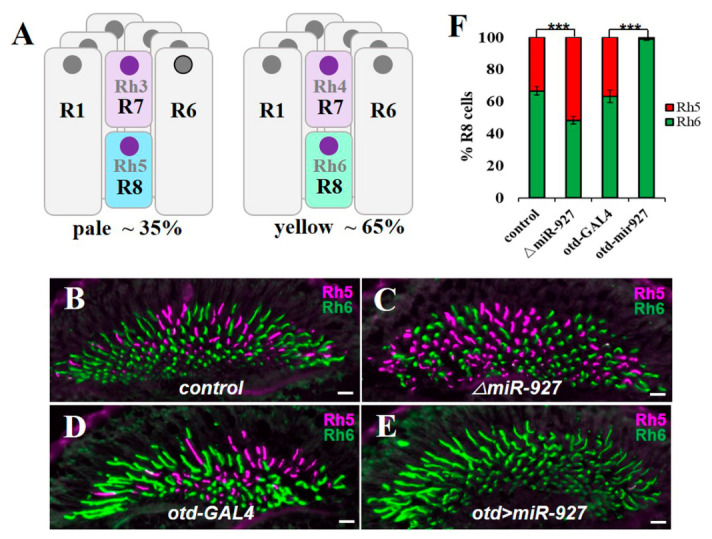
miR-927 regulates R8 photoreceptor subtype specification. (**A**) Schematic of *Drosophila* ommatidia showing pale (p) and yellow (y) subtypes. (**B**–**E**) Immunostaining of adult retinas showing Rh5 (red) and Rh6 (green) expression under the indicated genetic conditions. All scale bars, 10 µm. (**F**) Quantification of the proportions of Rh5- and Rh6-positive R8 photoreceptors in the indicated genotypes. *** *p* < 0.001. Error bars represent standard deviation. *n* = 8 retinas per genotype (~700 R8 cells).

Based on opsin expression, ommatidia are classified into two major subtypes. In pale (p) ommatidia, R7 cells express the UV-sensitive opsin, Rhodopsin 3 (Rh3), whereas R8 cells (pR8) express the blue-sensitive Rh5. In yellow (y) ommatidia, R7 cells express the long-wavelength UV-sensitive Rh4, and R8 cells (yR8) express the green-sensitive Rh6 [10,11,12]. These subtypes are stochastically distributed across the retina at an approximate ratio of 35:65 (p:y) and form functional R7–R8 pairs that enable wavelength discrimination [13,14,15,16] (Figure 1A).

R8 subtype identity is controlled by a Hippo pathway–dependent bistable regulatory circuit. In this system, the kinase Warts (Wts) and the PH-domain protein Melted (Melt) form a double-negative feedback loop that enforces mutually exclusive cell fates, resulting in either *Rh5* or *Rh6* expression [17,18,19,20]. In yR8 cells, active Wts inhibits the transcriptional co-activator Yorkie (Yki), thereby repressing *Rh5* expression and permitting *Rh6* expression. Conversely, in pR8 cells, reduced Wts activity allows Yki activation, promoting *Rh5* expression and suppressing *Rh6* fate [21,22,23]. At the transcriptional level, Hippo signaling regulates target gene expression through Yki and its DNA-binding partner Scalloped (Sd) [24,25,26].

However, transcriptional control alone cannot fully account for R8 subtype specification. Our previous work showed that the *Rh5* 3′ untranslated region (3′UTR) mediates post-transcriptional regulation of Rh5 expression, revealing an additional regulatory layer downstream of Hippo signaling [25]. Given that microRNAs act through 3′UTRs, this observation suggests that specific miRNAs may contribute to R8 subtype specification [27,28,29,30]. However, the identities of such miRNAs and the mechanisms by which they are integrated into the Hippo-dependent regulatory circuit remain unknown.

To address this question, we systematically analyzed the *Rh5* 3′UTR and identified miR-927 as a key regulator of R8 subtype fate. We show that miR-927 acts within R8 photoreceptors to promote yR8 identity while suppressing pR8 fate. Mechanistically, miR-927 directly represses both the Hippo pathway effector *yki* and the terminal differentiation gene *Rh5* through their 3′UTRs, thereby coupling pathway output to downstream gene expression. These findings reveal how post-transcriptional regulation is integrated into a bistable signaling network to modulate binary cell fate decisions.

## 2. Materials and Methods

### 2.1. Plasmid Construction and Generation of Transgenic Flies

The *pattB-pRh5-LacZ-3′UTR^SV40^* reporter was generated by subcloning the *Rh5* promoter fragment (*pRh5-269/+50*) into the *pattB-lacZ* vector [31]. The *pattB-melt450-nLacZ* reporter was described previously [17]. Mutations in Sd binding sites within the *Rh5* promoter were introduced by site-directed mutagenesis as described [25]. The *Rh5* and *yki* 3′UTRs were PCR-amplified and cloned downstream of the reporter constructs. Mutations in the predicted miR-927 binding sites within the 3′UTRs were generated by site-directed mutagenesis using the following primers, with mutated sequences indicated in the constructs. The constructs were integrated into the *Drosophila* genome using standard ΦC31-mediated transgenesis.

Rh5-3UTR1_forward: gatcCTCGAGtggtacaattgtcagattaacgaag
Rh5-3UTR1_reverse: gatcGGTACCtttaaataattttttaatgccaac
Rh5-3UTR2_forward: gtcagattaacgaagtgaaaactttaaatcatacg
Yki-3UTR1_forward: ataagaatGCGGCCGCttcaatgtatacatctgtattagacc
Yki-3UTR1_reverse: cggGGTACCcgtttatgtaaagaaatactataaaatttg
Yki-3UTR2_forward1: ttcaatgtatacatctgtattagacctaaaagttttatattttg
Yki-3UTR2_forward2: cctaaaagttttatattttgtattataattaaatatttttcaaattttatag
Yki-3UTR2_reverse: cgtttatgtaaagaaatactataaaatttgaaaaatatttaattataatac

### 2.2. Drosophila Stocks

The following fly stocks were used: *wts-nLacZ*, *sensR8-GAL4* [25]; *UAS-yki^RNAi^* (KK109756), *UAS-wts^RNAi^* (KK101055), *UAS-hpo^RNAi^* (KK101704), were obtained from the Vienna Drosophila Resource Center (VDRC) (Vienna, Austria). *otd-GAL4*, *sev-GAL4*, *UAS-Luciferase* (BDSC #35788), *miR-927^KO^* (BDSC #58935), *UAS-miR-927* (BDSC #60600), UAS-*miR-92a* (BDSC #59876), *miR-92a ^KO^* (BDSC #41153), *UAS-miR-310-313* cluster (BDSC #41135), *miR-310-313 ^KO^* (BDSC #7898 and BDSC #6609), *UAS-miR-92a* (BDSC #60674), *miR-iab8^KO^* (BDSC #1467) and *UAS-melt* (BDSC #1777) were obtained from the Bloomington Drosophila Stock Center (BDSC) (Bloomington, USA). Fly stocks were maintained using standard genetic procedures. Control genotypes were selected according to each experimental design. For loss-of-function experiments, w^1118^ was used as the control. For GAL4/UAS experiments, corresponding driver or UAS controls were included. The *otd-GAL4* driver is expressed in all R8 photoreceptors [32]. *UAS-luciferase* was used as a transgene dosage control in experiments involving co-expression of two UAS constructs. In these cases, control flies carried an additional UAS-luciferase transgene to match the total number of UAS elements present in experimental genotypes. For experiments involving a single UAS transgene, no additional dosage control was applied. Flies were raised on standard cornmeal–molasses media at 25 °C under a 12 h:12 h light–dark cycle.

### 2.3. Immunohistochemistry

Fly head dissection was performed as previously described [33]. Heads were embedded in OCT compound, frozen, and sectioned at 12 μm using a CM1850 cryostat (Leica, Wetzlar, Germany). Sections were fixed in 4% formaldehyde in PBS and washed three times for 10 min each in PBX (PBS containing 0.3% Triton X-100). Samples were incubated with primary antibodies overnight at 4 °C in BNXS buffer (1× PBX, 0.1% BSA, 0.05% saponin), followed by three 10 min washes in PBX. Secondary antibodies diluted in BNXS buffer were applied for 90 min at room temperature. After three additional washes in PBX, slides were mounted using Fluoromount (Sigma, Kawasaki, Kanagawa). Primary antibodies were used at the following dilutions: mouse anti-Rh5 (1:1000) [34]; rabbit anti-Rh6 (1:2000) [35]; mouse anti-Elav (1:100; DSHB, elav-9F8A9), and chicken anti-LacZ (1:1000; Abcam). Alexa Fluor 488-, 555-, and 647-conjugated secondary antibodies (1:1500; Invitrogen) were used. Images were acquired using an Apotome deconvolution system (Zeiss ApoTome.2; Carl Zeiss, Oberkochen, Germany). Images were collected under identical settings within each experiment and processed using Axiovision 4.5 (Carl Zeiss, Oberkochen, Germany). Elav-positive R8 photoreceptors were scored for Rh5, Rh6, or LacZ expression from at least three independent animals and two biological replicates. Statistical significance was determined using a two-tailed unpaired Student’s *t*-test.

### 2.4. miRNA Target Prediction

Candidate miRNAs targeting the *Rh5* 3′UTR were initially identified using the microRNA.org database (August 2010 release), which is based on the miRanda algorithm. Default parameters were applied, and candidate sites were selected based on canonical seed sequence complementarity.

To identify conserved miR-927 binding sites, the 3′UTRs of *Rh5* and *yki* were further analyzed using TargetScan (release 7.2). Predicted targets were selected based on conserved seed matches and context++ scores according to standard TargetScan criteria. The combined use of these tools improves confidence in miRNA target prediction.

## 3. Results

### 3.1. miR-927 Promotes yR8 Fate and Represses pR8 Identity

To identify microRNAs that regulate *Rh5* expression at the post-transcriptional level, we analyzed the *Rh5* 3′UTR using the microRNA.org database (based on the miRanda algorithm) and selected candidate miRNAs predicted to bind this region, including miR-92a, the miR-310–313 cluster, miR-iab8, and miR-927. We then systematically tested their roles in R8 subtype specification by examining Rh5 and Rh6 protein expression using immunostaining. Loss- or gain-of-function of most candidate miRNAs did not significantly alter the proportions of Rh5- and Rh6-positive photoreceptors, indicating that they are not required for R8 subtype specification under these conditions. Notably, overexpression of miR-iab8 strongly increased Rh6 expression; however, miR-iab8 loss-of-function mutants did not exhibit a detectable phenotype (Figure S1), suggesting that its role in R8 subtype specification is not supported by loss-of-function evidence and may depend on non-physiological or context-specific conditions.

In contrast, miR-927 exhibited a strong and reproducible effect on R8 subtype identity. Loss of miR-927 resulted in a marked increase in Rh5-positive cells, accompanied by a corresponding reduction in Rh6-positive cells compared to wild-type controls, indicating an expansion of pR8 fate at the expense of yR8 fate (Figure 1C,D). Conversely, overexpression of miR-927 driven by otd-GAL4 produced the opposite phenotype, with a strong reduction in Rh5-positive cells and a near-complete expansion of Rh6-positive yR8s relative to driver controls (Figure 1E,F). Quantification confirmed a significant shift in R8 subtype ratios upon miR-927 manipulation (Figure 1F). Together, these results identify miR-927 as a potent regulator that biases R8 subtype specification toward the yR8 fate.

### 3.2. miR-927 Represses Rh5 Expression Through a 3′UTR-Dependent Mechanism

**Figure 2:**
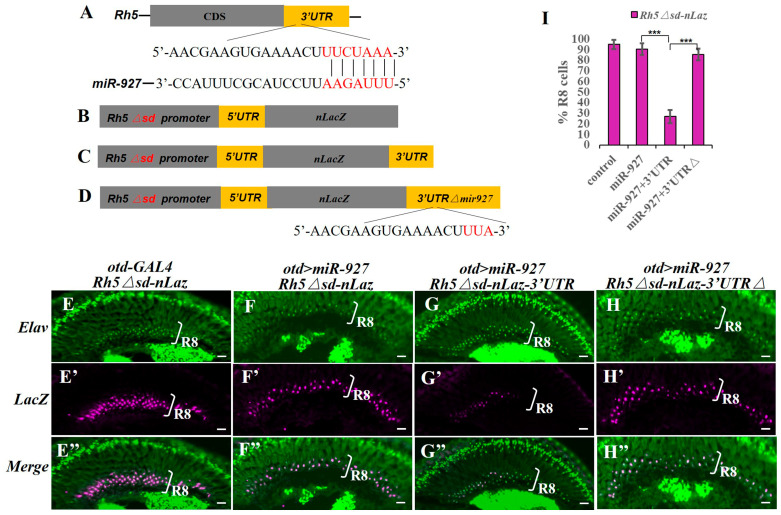
miR-927 directly represses *Rh5* via its 3′UTR. (**A**) Predicted miR-927 binding site within the *Rh5* 3′UTR, showing base pairing between miR-927 and the target sequence. (**B**,**C**) Schematic of LacZ reporters driven by a modified *Rh5* promoter lacking Sd binding sites. Reporters either lack the *Rh5* 3′UTR (**B**) or contain the *Rh5* 3′UTR (**C**). (**D**) Schematic of the *Rh5* 3′UTR reporter carrying point mutations in the predicted miR-927 seed-matching sequence. (**E**–**H″**) Immunostaining of adult retinas showing LacZ expression (red) and photoreceptor marker immunostained by Elav (green) under the indicated conditions. All scale bars, 10 µm. (**I**) Quantification of reporter-positive R8 photoreceptors under the indicated conditions. *** *p* < 0.001. Error bars represent standard deviation. *n* = 8 retinas per genotype (~700 R8 cells).

To determine whether miR-927 directly regulates *Rh5* expression, we analyzed the *Rh5* 3′UTR using TargetScan (release 7.2) and identified a conserved miR-927 seed-matching site (Figure 2A). To test its functional relevance, we generated LacZ reporters driven by a modified *Rh5* promoter in which Sd binding sites were mutated, thereby uncoupling reporter expression from Hippo-dependent transcription [17,25]. This design enabled selective assessment of post-transcriptional regulation mediated by the *Rh5* 3′UTR.

miR-927 overexpression had no detectable effect on a reporter lacking the *Rh5* 3′UTR (Figure 2B,E–F″), indicating that miR-927 does not regulate the reporter in the absence of the 3′UTR. In contrast, inclusion of the *Rh5* 3′UTR resulted in a strong reduction in reporter expression, indicating that miR-927–mediated repression requires this region (Figure 2C,G–G″).

To test whether repression depends on the predicted binding site, we introduced point mutations into the seed-matching sequence within the *Rh5* 3′UTR (Figure 2D). Disruption of this site abolished miR-927–dependent repression (Figure 2H–H″). Together, these results demonstrate that miR-927 directly represses *Rh5* expression through a specific binding site in its 3′UTR.

### 3.3. miR-927 Acts Cell-Autonomously in R8 Photoreceptors

The strong expansion of Rh6-positive cells upon miR-927 overexpression suggests that its function is not limited to repression of *Rh5*. Because R8 subtype specification is influenced by both intrinsic programs and R7-derived signals, we asked whether miR-927 acts cell-autonomously in R8 photoreceptors.

**Figure 3:**
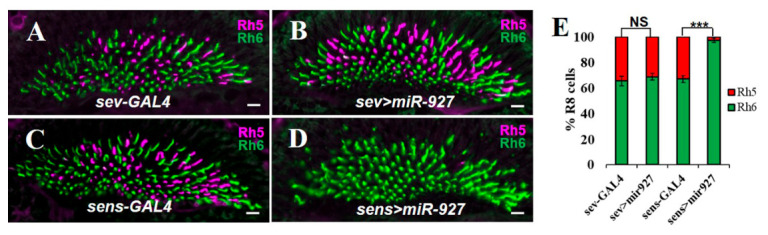
miR-927 functions cell-autonomously in R8 photoreceptors. (**A**,**B**) Rh5 (red) and Rh6 (green) expression in control (sev-GAL4) and R7-specific miR-927 overexpression (sev > miR-927). (**C**,**D**) Rh5 (red) and Rh6 (green) expression in control (sens-GAL4) and R8-specific miR-927 overexpression (sens > miR-927). All scale bars, 10 µm. (**E**) Quantification of Rh5- and Rh6-positive R8 photoreceptors. NS, not significant; *** *p* < 0.001. Error bars represent standard deviation. *n* = 8 retinas per genotype (~700 R8 cells).

To test this, we overexpressed miR-927 in R7 or R8 cells using cell type–specific drivers [17]. Expression of miR-927 in R7 cells (sev-GAL4) did not affect the distribution of Rh5- and Rh6-positive photoreceptors. In contrast, expression in R8 cells (sens-GAL4) resulted in a strong shift toward Rh6-positive yR8 cells, accompanied by a reduction in Rh5-positive pR8 cells (Figure 3A–D). These results indicate that miR-927 functions cell-autonomously within R8 photoreceptors to control subtype specification.

### 3.4. miR-927 Regulates Wts and Melt Expression in R8 Photoreceptors

**Figure 4:**
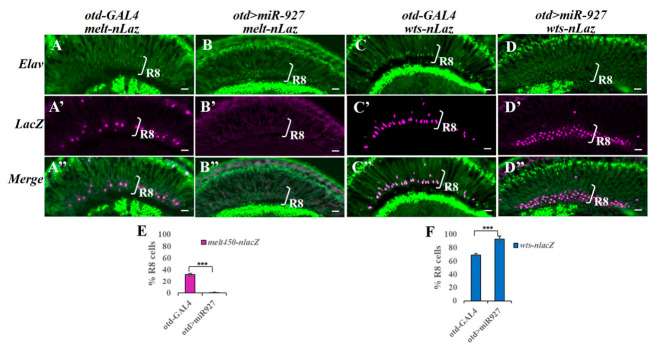
miR-927 regulates the expression of *wts* and *melt*. (**A**–**D″**) Expression of *melt450-nLacZ* (red; **A**–**B″**) and wts-nLacZ (red; **C**–**D″**) in control (otd-GAL4) and miR-927-overexpressing (otd > miR-927) retinas. Photoreceptors are labeled by Elav (green). All scale bars, 10 µm. (**E**,**F**) Quantification of Melt- and Wts-positive R8 photoreceptors under the indicated conditions. *** *p* < 0.001; Error bars represent standard deviation. *n* = 8 retinas per genotype (~700 R8 cells).

R8 subtype specification is controlled by a bistable circuit in which Melt and Wts are expressed in mutually exclusive patterns, marking pR8 and yR8 fates, respectively. To determine whether miR-927 modulates this circuit, we examined *melt* and *wts* expression using a *melt* reporter (*melt450-nLacZ*) and a *wts* enhancer trap (*wts-nLacZ*) [17,36]. In control retinas, *melt* was restricted to a subset of R8 cells, whereas *wts* was expressed in the complementary population. Upon miR-927 overexpression, *melt* expression was strongly reduced, while wts expression expanded to the majority of R8 cells (Figure 4A–D″). Quantification confirmed a marked shift in the proportions of Melt- and Wts-positive cells (Figure 4E,F). These results indicate that miR-927 biases the Melt–Wts circuit toward the yR8 state. These effects are consistent with modulation of the Hippo pathway–dependent bistable circuit involving Melt and Wts, although the underlying mechanism remains unclear.

### 3.5. miR-927 Acts Downstream of Hippo Pathway Core Components

**Figure 5:**
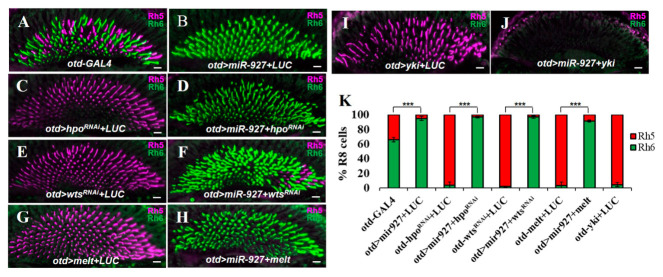
miR-927 acts downstream of the Hippo pathway core components. (**A**–**H**) Rh5 (red) and Rh6 (green) expression in control and indicated genetic backgrounds. (**I**,**J**) Rh5 and Rh6 expression in retinas overexpressing yki alone or together with miR-927. All scale bars, 10 µm. (**K**) Quantification of Rh5- and Rh6-positive R8 photoreceptors. In co-expression experiments, UAS-luciferase was included in control genotypes to balance UAS transgene dosage. *** *p* < 0.001; Error bars represent standard deviation. *n* = 8 retinas per genotype (~700 R8 cells).

To position miR-927 within the Hippo signaling hierarchy, we performed epistasis analyses with core pathway components. As expected, knockdown of *hpo* or *wts*, or overexpression of *melt*, strongly shifted R8 fate toward Rh5-positive pR8 cells at the expense of Rh6-positive yR8 cells (Figure 5C,E,G). Strikingly, co-expression of miR-927 in these backgrounds modifies the phenotype, as R8 cells remained biased toward yR8 identity (Figure 5D,F,H). These results indicate that miR-927 does not act upstream of the Hippo pathway core kinase cascade.

To further examine the relationship between miR-927 and Hippo pathway output, we examined its genetic relationship with the transcriptional co-activator *yki*. Overexpression of *yki* alone led to a strong expansion of Rh5-positive pR8 fate, consistent with its role in promoting pR8 fate (Figure 5I). However, co-expression of miR-927 with *yki* resulted in the loss of both Rh5 and Rh6 expression (Figure 5J). The loss of Rh5 is consistent with direct repression by miR-927, whereas the absence of Rh6 likely reflects the effect of constitutive Yki activity on subtype specification. Notably, the UAS-yki transgene used in this experiment lacks the endogenous 3′UTR and is therefore not subject to miRNA-mediated regulation. Thus, this experimental configuration does not reflect the endogenous regulatory relationship between miR-927 and yki, and the observed phenotype should be interpreted as a non-physiological outcome of combined overexpression rather than direct epistasis within the native Hippo signaling hierarchy.

### 3.6. miR-927 Represses yki Through Its 3′UTR

**Figure 6:**
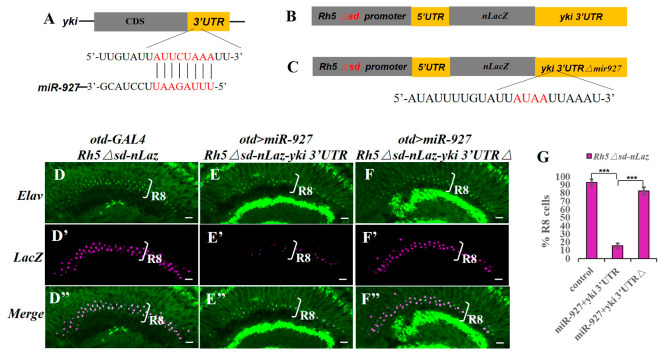
miR-927 directly represses *yki* via its 3′UTR. (**A**) Predicted miR-927 binding site within the *yki* 3′UTR, showing base pairing between miR-927 and the target sequence. (**B**) Schematic of a LacZ reporter driven by a modified *Rh5* promoter lacking Sd binding sites, with the *yki* 3′UTR inserted downstream. (**C**) Schematic of the reporter carrying mutations in the miR-927 seed-matching site within the *yki* 3′UTR. (**D**–**F″**) Expression of LacZ (red) in control and miR-927-overexpressing retinas. Photoreceptors are labeled by Elav (green). All scale bars, 10 µm. (**G**) Quantification of reporter-positive R8 photoreceptors under the indicated conditions. *** *p* < 0.001. Error bars represent standard deviation. *n* = 8 retinas per genotype (~700 R8 cells).

To assess whether miR-927 can regulate yki through its 3′UTR, we analyzed the *yki* 3′UTR using TargetScan (release 7.2) and identified a conserved miR-927 seed-matching site (Figure 6A). To assess its functional relevance, we generated LacZ reporters driven by a modified *Rh5* promoter lacking Sd binding sites, thereby uncoupling reporter expression from Hippo-dependent transcription. The *yki* 3′UTR was inserted downstream of the reporter to enable selective analysis of post-transcriptional regulation (Figure 6B). In control retinas, the reporter showed robust LacZ expression in R8 photoreceptors (Figure 6D–D″). miR-927 overexpression strongly reduced reporter expression, indicating repression through the *yki* 3′UTR (Figure 6E–E″). To determine whether this repression depends on the predicted binding site, we introduced mutations into the seed-matching sequence within the *yki* 3′UTR (Figure 6C). Disruption of this site abolished miR-927–mediated repression, restoring LacZ expression to control levels (Figure 6F–F″). These results demonstrate that miR-927 can repress *yki* expression via its 3′UTR.

## 4. Discussion

Binary cell fate decisions rely on regulatory architectures that convert graded or variable signaling inputs into stable and mutually exclusive gene expression states [37,38,39]. In the *Drosophila* retina, R8 photoreceptor subtype specification provides a well-defined system in which such mechanisms can be dissected. Here, we identify miR-927 as a post-transcriptional regulator that interfaces with the Hippo pathway to bias R8 subtype fate.

**Figure 7:**
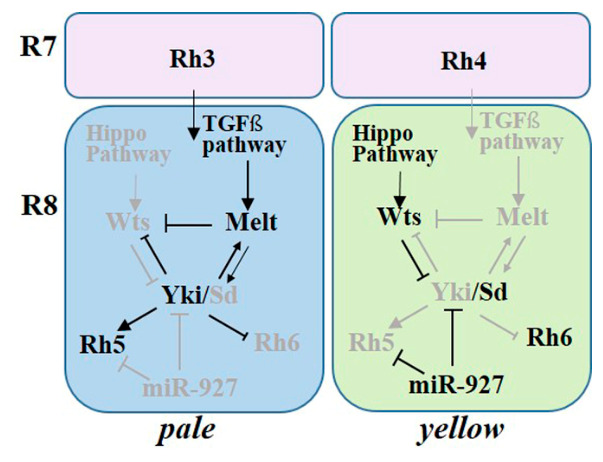
Model for miR-927 function in R8 photoreceptor subtype specification. Schematic model of R8 subtype specification in pale (p) and yellow (y) ommatidia. In pR8 cells, Yki/Sd activity promotes Rh5 expression, while in yR8 cells, Wts inhibits Yki, allowing Rh6 expression. miR-927 biases this system toward yR8 fate by repressing Rh5 and potentially modulating yki via its 3′UTR. Arrows indicate activation, and blunt-ended lines indicate repression. Gray elements represent inactive components.

Our results demonstrate that miR-927 promotes yR8 identity while suppressing pR8 fate. Mechanistically, miR-927 directly represses the terminal differentiation gene *Rh5* and can repress the Hippo pathway effector *yki* through their 3′UTRs. By simultaneously repressing a signaling effector and its downstream output, miR-927 may establish a coherent regulatory relationship between pathway activity and terminal gene expression. Notably, targeting both *yki* and *Rh5* may constitute a feed-forward regulatory architecture, in which repression of a signaling effector and its downstream target reinforces a consistent cell fate outcome (Figure 7).

However, while our reporter assays demonstrate that miR-927 can repress yki expression via its 3′UTR, the endogenous spatial relationship between miR-927 and Yki in R8 photoreceptors remains to be determined. In particular, whether miR-927 expression is inversely correlated with Yki activity in vivo is currently unknown. Therefore, the observed regulation should be interpreted as evidence for the potential of miR-927 to modulate yki, rather than a definitive demonstration of physiological interaction. Future studies combining miRNA detection approaches (e.g., smFISH) with Yki protein analysis will be important to clarify this relationship.

The Hippo pathway controls R8 subtype specification through a bistable circuit involving Wts and Melt. Genetic epistasis analyses further indicate that miR-927 acts at the level of Hippo pathway output. Notably, miR-927 overrides the effects of perturbations in core pathway components, suggesting that post-transcriptional regulation can dominate over upstream signaling inputs in determining cell fate. Within this Hippo pathway dependent regulatory framework, Yki activity is associated with pR8 fate, whereas Wts activity defines yR8 identity as part of a bistable circuit involving Melt and Wts [17,18]. However, direct transcriptional regulation of *melt* or *wts* by Yki has not been clearly established. Therefore, the relationships among these components should be interpreted within the context of pathway-level regulatory interactions rather than direct gene regulation. However, it should be noted that the yki overexpression experiment was performed using a UAS-yki transgene lacking its endogenous 3′UTR, which is therefore refractory to miR-927-mediated repression. As a result, the observed phenotype likely reflects the combined effects of miR-927 and constitutive Yki activity, rather than a strict epistatic relationship.

miR-927 overexpression leads to strong repression of Melt expression. However, bioinformatic analysis did not identify putative miR-927 binding sites within the *melt* transcript, indicating that *melt* is unlikely to be a direct target. Instead, the observed repression is likely mediated indirectly through upstream regulatory components. Given that Yki positively regulates Melt expression, inhibition of *yki* by miR-927 provides a plausible explanation for the reduced Melt levels observed in our experiments. Further investigation will be required to determine whether additional indirect pathways or unidentified targets contribute to this regulation. These findings reveal an additional layer of control in which microRNAs modulate not only downstream targets but also the effective output of signaling pathways.

Despite its strong effect, loss of miR-927 does not eliminate yR8 fate but instead shifts subtype proportions toward a more balanced distribution. This indicates that miR-927 is not an essential determinant of R8 fate, but rather a biasing factor that stabilizes subtype asymmetry. Consistent with this interpretation, our previous work showed that eye-specific disruption of miRNA biogenesis has minimal effects on R8 subtype ratios [25], suggesting that robustness in this system arises from the combined activity of multiple miRNAs. In this context, individual miRNAs likely act in a combinatorial manner to fine-tune cell fate outcomes. Our preliminary analyses further suggest that some miRNAs may exert opposing effects on R8 subtype specification (unpublished), although their roles remain to be fully characterized.

Within this framework, the interpretation of the miR-iab8 phenotype requires caution. Although overexpression of miR-iab8 strongly biases R8 fate toward the yR8 subtype, loss-of-function mutants do not exhibit a detectable phenotype. One possible explanation is that miR-iab8 is not endogenously expressed in R8 photoreceptors, limiting the physiological relevance of both gain- and loss-of-function analyses. Alternatively, functional redundancy among microRNAs may mask its loss-of-function phenotype. Thus, miR-iab8 may represent a context-dependent regulator whose contribution becomes apparent only under specific conditions or in combination with other miRNAs.

The role of miR-927 in Hippo pathway regulation is likely not restricted to the retina. Previous studies have shown that miR-927 regulates wing development by targeting the Hippo pathway [40], suggesting that it may function as a context-independent modulator of Hippo signaling. Together with our findings, this supports a model in which microRNAs act at the level of pathway output to influence cell fate decisions across tissues.

## 5. Conclusions

In this study, we identify miR-927 as a post-transcriptional regulator that biases R8 photoreceptor subtype specification in *Drosophila*. miR-927 promotes yR8 identity while repressing pR8 fate, thereby modulating the balance between Rh5- and Rh6-expressing photoreceptors.

Mechanistically, miR-927 directly represses the terminal differentiation gene Rh5 and is capable of targeting the Hippo pathway effector yki through their 3′UTRs. This dual regulation links upstream signaling output to terminal gene expression, providing a mechanism for integrating post-transcriptional control into a bistable regulatory system.

Furthermore, our findings suggest that microRNAs can function as modulators of signaling pathway output rather than primary determinants of cell fate. By biasing and stabilizing subtype identity, miR-927 contributes to the robustness of binary cell fate decisions.

## Data Availability

The original contributions presented in this study are included in the article/Supplementary Materials. Further inquiries can be directed to the corresponding authors.

## Supplementary Materials

The following supporting information can be downloaded at: https://www.mdpi.com/article/10.3390/cells15090841/s1, Figure S1: Quantification of R8 photoreceptor subtype distribution upon manipulation of candidate miRNAs. Bar plots showing the proportions of Rh5- and Rh6-positive R8 photoreceptors following gain-of-function (otd-GAL4-driven overexpression) or loss-of-function (mutant) of miR-92a, the miR-310–313 cluster, and miR-iab8. otd-GAL4 was used as the control for overexpression and w^1118^ was used as the control for loss-of-function experiments. ***p* < 0.001. Error bars represent s.d. Bars without significance labels are not significantly different from the corresponding controls.

## References

[1] Schnaitmann C., Pagni M., Reiff D.F. (2020). Color vision in insects: Insights from *Drosophila*. J. Comp. Physiol. A Neuroethol. Sens. Neural Behav. Physiol..

[2] Osorio D., Vorobyev M. (2005). Photoreceptor spectral sensitivities in terrestrial animals: Adaptations for luminance and colour vision. Proc. Biol. Sci..

[3] Kelber A., Vorobyev M., Osorio D. (2003). Animal colour vision--behavioural tests and physiological concepts. Biol. Rev. Camb. Philos. Soc..

[4] Hardie R.C., Raghu P. (2001). Visual transduction in *Drosophila*. Nature.

[5] Schnaitmann C., Haikala V., Abraham E., Oberhauser V., Thestrup T., Griesbeck O., Reiff D.F. (2018). Color Processing in the Early Visual System of *Drosophila*. Cell.

[6] Cook T., Desplan C. (2001). Photoreceptor subtype specification: From flies to humans. Semin. Cell Dev. Biol..

[7] Viets K., Eldred K., Johnston R.J. (2016). Mechanisms of Photoreceptor Patterning in Vertebrates and Invertebrates. Trends Genet..

[8] Carthew R.W. (2007). Pattern formation in the *Drosophila* eye. Curr. Opin. Genet. Dev..

[9] Zhang X., Shinjo R., Kitamata M., Otsune S., Nakagoshi H. (2026). Gap junction-mediated signaling coordinates Rhodopsin coupling for *Drosophila* color vision. Biol. Open.

[10] Wernet M.F., Mazzoni E.O., Celik A., Duncan D.M., Duncan I., Desplan C. (2006). Stochastic spineless expression creates the retinal mosaic for colour vision. Nature.

[11] Mollereau B., Domingos P.M. (2005). Photoreceptor differentiation in *Drosophila*: From immature neurons to functional photoreceptors. Dev. Dyn..

[12] Schnaitmann C., Garbers C., Wachtler T., Tanimoto H. (2013). Color discrimination with broadband photoreceptors. Curr. Biol..

[13] Hardie R.C. (2001). Phototransduction in *Drosophila melanogaster*. J. Exp. Biol..

[14] Kitamata M., Otake Y., Kitagori H., Zhang X., Maki Y., Boku R., Takeuchi M., Nakagoshi H. (2024). Functional opsin patterning for *Drosophila* color vision is established through signaling pathways in adjacent object-detection neurons. Development.

[15] Heath S.L., Christenson M.P., Oriol E., Saavedra-Weisenhaus M., Kohn J.R., Behnia R. (2020). Circuit Mechanisms Underlying Chromatic Encoding in *Drosophila* Photoreceptors. Curr. Biol..

[16] Ordway A.J., Helt R.N., Johnston R.J. (2024). Transcriptional priming and chromatin regulation during stochastic cell fate specification. Philos. Trans. R. Soc. Lond. B Biol. Sci..

[17] Jukam D., Xie B., Rister J., Terrell D., Charlton-Perkins M., Pistillo D., Gebelein B., Desplan C., Cook T. (2013). Opposite feedbacks in the Hippo pathway for growth control and neural fate. Science.

[18] Mikeladze-Dvali T., Wernet M.F., Pistillo D., Mazzoni E.O., Teleman A.A., Chen Y.W., Cohen S., Desplan C. (2005). The growth regulators warts/lats and melted interact in a bistable loop to specify opposite fates in *Drosophila* R8 photoreceptors. Cell.

[19] Pojer J.M., Saiful Hilmi A.J., Kondo S., Harvey K.F. (2021). Crumbs and the apical spectrin cytoskeleton regulate R8 cell fate in the *Drosophila* eye. PLoS Genet..

[20] Rader A.E., Bayarmagnai B., Frolov M.V. (2023). Combined inactivation of RB and Hippo converts differentiating *Drosophila* photoreceptors into eye progenitor cells through derepression of *homothorax*. Dev. Cell.

[21] Misra J.R., Irvine K.D. (2018). The Hippo Signaling Network and Its Biological Functions. Annu. Rev. Genet..

[22] Huang J., Wu S., Barrera J., Matthews K., Pan D. (2005). The Hippo signaling pathway coordinately regulates cell proliferation and apoptosis by inactivating Yorkie, the *Drosophila* Homolog of YAP. Cell.

[23] Bunker J., Bashir M., Bailey S., Boodram P., Perry A., Delaney R., Tsachaki M., Sprecher S.G., Nelson E., Call G.B. (2023). Blimp-1/PRDM1 and Hr3/RORbeta specify the blue-sensitive photoreceptor subtype in *Drosophila* by repressing the hippo pathway. Front. Cell Dev. Biol..

[24] Manning S.A., Kroeger B., Deng Q., Brooks E., Fonseka Y., Hinde E., Harvey K.F. (2024). The *Drosophila* Hippo pathway transcription factor Scalloped and its co-factors alter each other’s chromatin binding dynamics and transcription in vivo. Dev. Cell.

[25] Xie B., Morton D.B., Cook T.A. (2019). Opposing transcriptional and post-transcriptional roles for Scalloped in binary Hippo-dependent neural fate decisions. Dev. Biol..

[26] Goulev Y., Fauny J.D., Gonzalez-Marti B., Flagiello D., Silber J., Zider A. (2008). SCALLOPED interacts with YORKIE, the nuclear effector of the hippo tumor-suppressor pathway in *Drosophila*. Curr. Biol..

[27] Zhukova M., Schedl P., Shidlovskii Y.V. (2024). The role of secondary structures in the functioning of 3′ untranslated regions of mRNA: A review of functions of 3′ UTRs’ secondary structures and hypothetical involvement of secondary structures in cytoplasmic polyadenylation in *Drosophila*. Bioessays.

[28] Bartel D.P. (2009). MicroRNAs: Target recognition and regulatory functions. Cell.

[29] Colaianni D., De Pitta C. (2022). The Role of microRNAs in the *Drosophila* Melanogaster Visual System. Front. Cell Dev. Biol..

[30] Jang D., Kim C.J., Shin B.H., Lim D.H. (2024). The Biological Roles of microRNAs in *Drosophila* Development. Insects.

[31] Bischof J., Maeda R.K., Hediger M., Karch F., Basler K. (2007). An optimized transgenesis system for *Drosophila* using germ-line-specific phiC31 integrases. Proc. Natl. Acad. Sci. USA.

[32] Johnston R.J., Otake Y., Sood P., Vogt N., Behnia R., Vasiliauskas D., McDonald E., Xie B., Koenig S., Wolf R. (2011). Interlocked feedforward loops control cell-type-specific Rhodopsin expression in the *Drosophila* eye. Cell.

[33] Cook T., Pichaud F., Sonneville R., Papatsenko D., Desplan C. (2003). Distinction between color photoreceptor cell fates is controlled by Prospero in *Drosophila*. Dev. Cell.

[34] Chou W.H., Hall K.J., Wilson D.B., Wideman C.L., Townson S.M., Chadwell L.V., Britt S.G. (1996). Identification of a novel *Drosophila* opsin reveals specific patterning of the R7 and R8 photoreceptor cells. Neuron.

[35] Tahayato A., Sonneville R., Pichaud F., Wernet M.F., Papatsenko D., Beaufils P., Cook T., Desplan C. (2003). Otd/Crx, a dual regulator for the specification of ommatidia subtypes in the *Drosophila* retina. Dev. Cell.

[36] Sang Q., Wang G., Morton D.B., Wu H., Xie B. (2021). The ZO-1 protein Polychaetoid as an upstream regulator of the Hippo pathway in *Drosophila*. PLoS Genet..

[37] Ferrell J.E. (2002). Self-perpetuating states in signal transduction: Positive feedback, double-negative feedback and bistability. Curr. Opin. Cell Biol..

[38] Xiong W., Ferrell J.E. (2003). A positive-feedback-based bistable ‘memory module’ that governs a cell fate decision. Nature.

[39] Sagner A., Briscoe J. (2017). Morphogen interpretation: Concentration, time, competence, and signaling dynamics. Wiley Interdiscip. Rev. Dev. Biol..

[40] Yu X., Sun B., Gao X., Liu Q., Zhou Z., Zhao Y. (2025). miR-927 regulates insect wing development by targeting the Hippo pathway. Insect Sci..

