# Supplementary figures and images for: miR-927 Regulates Photoreceptor Subtype Specification Through Yorkie and Sensory Opsins in Drosophila
Source: Cells. 2026 May 4;15(9):841. doi: 10.3390/cells15090841 (PMC13162797; doi:10.3390/cells15090841)

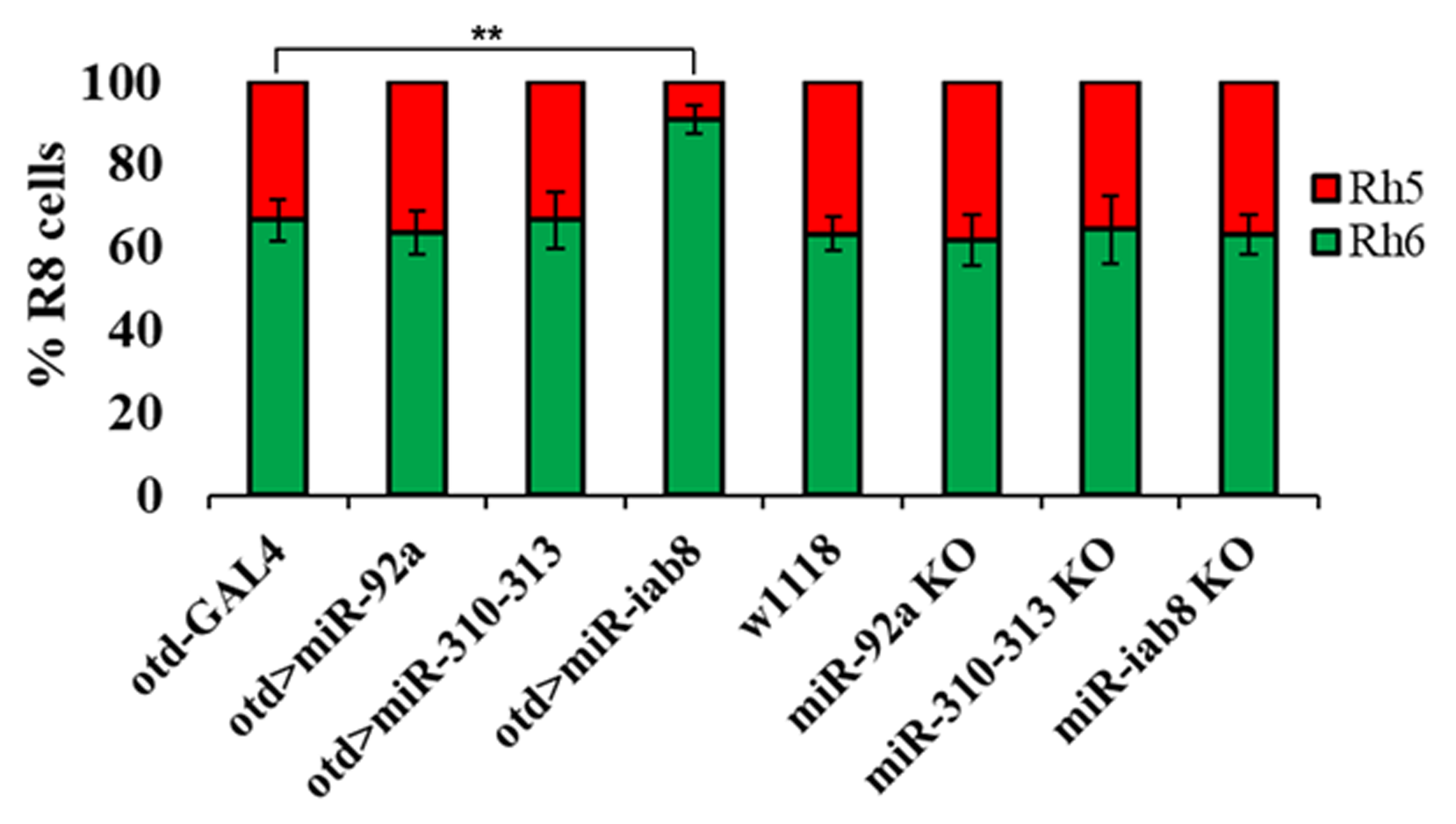

Supplement: Supplementary file 1 [file cells-15-00841-s001.zip › Figure S1.tif]
